# Genito-Urinary Surgery

**Published:** 1906-04-14

**Authors:** 


### GENITO-URINARY SURGERY.

(*Continued from Vol. XXXIX., page 425.*)

**Renal Traumatism.**—C. G. Cumston (Boston, Mass.),[5] in discussing this subject, points out that it is far more frequently met with in the male than the female. Thus in Kuster's statistics 94 per cent. of the patients were males. In the majority of cases the diagnosis can be made with a fair amount of certainty from the symptoms. These are general and local. The general symptoms include apathy immediately after the accident, followed or not by unconsciousness. Sometimes syncope of several days' duration supervenes. Vomiting, a small and frequent pulse, and subnormal temperature, are also symptomatic, and extreme pallor of the integuments may result from shock or internal hæmorrhage. The author found marked anæmia to be the most important general symptom. But the above general symptoms may arise in any accident, particularly in contusion of the abdomen, and frequently they do not correspond in any way with the real gravity of the accident. The local symptoms include severe local pain accompanied by tumefaction of the renal region and dullness on percussion, and hæmaturia. The pain is often colicky, like that due to the passage of a renal calculus. If the capsule is torn or the renal pelvis lacerated, effused fluid may pass into the retroperitoneal tissue or into the peritoneum. Abdominal distension usually masks the swelling at first, but when the abdominal walls become relaxed a tumour can be felt. Frequently the hæmaturia subsides soon after the injury, so that in a few days no blood elements are found in the urine. In some cases, however, with extensive laceration, the hæmaturia may last for weeks. When the peritoneum is lacerated and aseptic urine enters the peritoneal cavity, peritonitis does not necessarily result. Petroff collected 14 cases in which this had occurred, and in only one of these did septic peritonitis ensue. It is thus evident that one cannot state how often urine does pass into the peritoneum after renal injuries. If a perinephritic abscess results from infection the patient's life is endangered. With regard to treatment the author believes it is best to carefully watch the case, but to be ready to operate if the symptoms of severe hæmorrhage or signs of pus formation arise. T. C. Litler Jones [6] records a case in which a nephrectomy was performed for ruptured kidney in a man a few hours after an accident. The indications for operation were increasing dullness in the flank, a rising pulse-rate, and unbearable pain. A rib was found lower than that which had been regarded as the twelfth, which had no connection with the intercostal muscles. Probably it was a lumbar rib, and had been driven inwards into the kidney. The kidney was split into two parts. The lower third of the organ, with a portion of the pelvis attached, came away in the clots of blood which had collected. The patient recovered. Mr. Jones remarks that the first nephrectomy for ruptured kidney was performed by Dr. Rowdon in Liverpool. It was done 17 days after injury of the kidney on the right side. The child, however, died from pyelitis of the other kidney, illustrating the danger of delay in cases which have become septic.

**Acute Ascending Infection of the Urinary Tract.** H. L. Barnard [7] records two instances of this mode of infection of the kidneys which have been met with in the London Hospital, and also quotes the records of four gathered from literature. He sketches the symptoms as follows:—A woman, or less frequently a man, is suddenly attacked with severe scalding pain on micturition and frequency. Strangury follows The urine is turbid with leucocytes and epithelium, but remains acid or neutral. Pyrexia follows. On the second or third day the kidney becomes involved, revealed by pain and rigidity in the upper part of the abdomen and loin, rigors, vomiting, and the appearance of casts and more albumen in the urine than can be accounted for by the pus. The symptoms of cystitis and urethritis abate in a few days, and the renal signs become dominant. A tumour appears in the region of the infected kidney, and the abdominal wall is rigid and tender. In all the cases collected the right kidney was the one involved, and several times appendix trouble was wrongly suspected to exist. The condition of the kidney in the author's case is described as follows:—It was of twice its normal size, and intensely congested. The surface was covered with a great number of subcapsular hæmorrhagic spots, the larger ones with yellow centres. On dividing the organ the spots on the surface were seen to be the bases of wedge-shaped hæmorrhagic areas running into the pyramids. Microscopically the inflammation was found to be in the bundles of tubules forming the lobules, not around the vessels between the lobules, and it appeared to have started in the tubules. The appearances, together with the history, seemed to leave no doubt that there had been an acute ascending inflammation of the urinary tract. When an embolus is the cause of an infarct the wedge-shaped mass is interlobular, and the apex terminates between the pyramids, not in them as in these cases. In Lennander's case a pure culture of the colon bacillus was obtained from the kidney. It thus appears that in exceptional circumstances colon bacilli of very virulent type may ascend the normal urinary tract and produce as they go urethritis, cystitis, pyelitis, and finally multiple hæmorrhagic abscesses in the kidneys. If only one kidney is affected, and this is promptly removed, the patients quickly recover, and the infection in the lower organs is transient. Lennander has recorded five cases of acute renal infection with a formation of abscesses, which differed from those just described in that there was pre-existing obstructive disease in the lower urinary passages, and this group of cases is interesting as a connecting link between the rare cases of infection of the normal urinary tract and the form of infection which produces ordinary surgical kidney and is preceded by dilatation of the urinary tract. Treatment of the cases under consideration, if one kidney only is involved, which was the case in all the examples recorded, may be by nephrectomy, and this is the safest procedure. But in two of the six cases recovery followed, splitting and partially resecting the kidney and draining. The author concludes his paper with a discussion of the general pathology of renal infections by the three possible paths—the blood-vessels, the lymphatics, and the urinary passages.

**Hypernephroma.**—Taylor,[8] reports a case of this form of tumour in connection with the left kidney. The tumour and kidney were removed, and weighed together 6½ lbs. The patient recovered from the operation, but died within six months with recurrence. At the time of the patient's death there was great enlargement of the liver, and it was thought that the right kidney was implicated. The microscopical findings in the original tumour were typical of hypernephroma. The three important symptoms usually present in cases of hypernephroma are hæmaturia, renal colic, and the presence of a tumour, but in the present case the important symptom of hæmaturia was wanting. In some cases the tumour is intensely malignant, and removal is followed by rapid recurrence. Pathologists seem unable to distinguish the malignant from the non-malignant cases. One authority has stated that the symptom of hæmaturia means beginning degeneracy or malignancy, and that during the pre-malignant stage there is not likely to be hæmorrhage, but this does not hold true for the present case.

[6] Prac., Dec., 1905, p. 795. [6] Lancet, Sept. 23, 1905, p. 891. [7] Lancet, Oct. 28, 1905, p. 1243.

(*To be continued.*)

